# Methylation of *FAM110C* is a synthetic lethal marker for ATR/CHK1 inhibitors in pancreatic cancer

**DOI:** 10.2478/jtim-2023-0128

**Published:** 2024-07-27

**Authors:** Fengna Liu, Aiai Gao, Meiying Zhang, Yazhuo Li, Fan Zhang, James G. Herman, Mingzhou Guo

**Affiliations:** Department of Gastroenterology and Hepatology, the First Medical Center, Chinese PLA General Hospital, Beijing 100853, China; Department of Pathology, The Fourth Medical Center of PLA General Hospital, Beijing 100048, China; The Third Clinical College of Xinxiang Medical University, Xinxiang, Henan 453003, China; The Hillman Cancer Center, University of Pittsburgh Cancer Institute, Pittsburgh, PA 15213, USA; National Key Laboratory of Kidney Diseases, the First Medical Center, Chinese PLA General Hospital, Beijing 100853, China

**Keywords:** *FAM110C*, DNA methylation, pancreatic cancer, DNA damage repair, synthetic lethality

## Abstract

**Background and objectives:**

Pancreatic ductal adenocarcinoma (PDAC) is one of the deadliest malignancies. An epigenetic-based synthetic lethal strategy provides a novel opportunity for PDAC treatment. Finding more DNA damage repair (DDR)-related or cell fate-related molecules with aberrant epigenetic changes is becoming very important. Family with sequence similarity 110C (*FAM110C*) is a cell fate-related gene and its function in cancer remains unclear.

**Methods:**

Seven cell lines, 34 cases of intraductal papillary mucinous neoplasm (IPMN), 15 cases of mucinous cystic neoplasm (MCN) and 284 cases of PDAC samples were employed. Methylation-specific PCR, western blot, CRISPR knockout, immunoprecipitation and a xenograft mouse model were used in this study.

**Results:**

*FAM110C* is methylated in 41.18% (14/34) of IPMN, 46.67% (7/15) of MCN and 72.89% (207/284) of PDAC, with a progression trend from IPMN/MCN to pancreatic cancer (*P* = 0.0001, *P* = 0.0389). *FAM110C* methylation is significantly associated with poor overall survival (OS) (*P* = 0.0065) and is an independent prognostic marker for poor OS (*P* = 0.0159). *FAM110C* inhibits PDAC cells growth both *in vitro* and *in vivo*, serving as a novel tumor suppressor. *FAM110C* activates ATM and NHEJ signaling pathways by interacting with HMGB1. Loss of *FAM110C* expression sensitizes PDAC cells to VE-822 (an ATR inhibitor) and MK-8776 (a CHK1 inhibitor).

**Conclusion:**

*FAM110C* methylation is a potential diagnostic and prognostic marker in PDAC, and its epigenetic silencing sensitizes PDAC cells to ATR/CHK1 inhibitors.

## Introduction

Pancreatic ductal adenocarcinoma cancer (PDAC) is the most malignant tumor and is expected to be the second leading cause of cancer-related death by 2030.^[1,2]^ There are three major PDAC precursor lesions, including intraductal papillary mucinous neoplasm (IPMN), pancreatic intraepithelial neoplasia (PanIN) and mucinous cystic neoplasm (MCN).^[3,4]^ With the extensive application of next generation sequencing, precision medicine has been growing very fast in oncology. However, unlike other types of cancer, the development of tailored therapeutics for PDAC has been largely unsatisfactory. Oncogenetic mutations of *KRAS* account for 95% of PDAC patients, yet targeting their proteins is challenging due to the high affinity for GTP and/or ADP.^[5,6]^ Despite the promising development of a *KRAS*^G12C^ inhibitor in preclinical models,^[7]^ it is important to note that this specific mutation accounts for only approximately 1% of all *KRAS* mutations.^[8]^ Due to the high levels of intratumoral and intertumoral heterogeneity as well as the presence of untargetable mutations in *TP53*, *SMAD4*, and *CDKN2A/B* genes, precision medicine in pancreatic cancer is still unapplicable.^[5,9,10]^ *BRCA1/2* mutations and other DNA damage repair (DDR) gene mutations provide new opportunities for PADC therapy by applying a synthetic lethal strategy.^[11]^ Nevertheless, most of these genes are mutated by less than 5%, including *BRCA1/2*, *PALB2*, *ATM* and *MLH1*,^[6]^ necessitating the search for additional actionable targets.

With exhaustive genomic resources, aberrant epigenetic changes may provide more avenues for cancer therapy. The classical epi-drugs primarily focus on targeting epigenetic regulators, including readers, writers, and erasers.^[12]^ The ideal cancer therapeutic strategy is to target aberrantly changed cancer cells precisely, without damaging normal cells. Synthetic lethality can be employed by leveraging abnormal epigenetic changes to selectively eliminate cancer cells. For this purpose, the aberrant epigenetic changes of key components in DDR or cell fate-related pathways need to be identified.

Family with sequence similarity 110 (*FAM110*) family has been identified, consisting of *FAM110A*, *B*, *C* and *D*.^[13,14]^ FAM110 proteins were found to be located to centrosomes and accumulated at the microtubule organization center in interphase and at spindle poles during mitosis.^[14]^ The data for the *FAM 110* family in cancer is very limited.^[15,16]^ *FAM 110C* protein was reported to inhibit cell proliferation by inducing G1/S arrest.^[14,17]^ Additionally, *FAM 110C* was found to interact with the microtubule cytoskeleton and suppress cell migration by inhibiting AKT signaling.^[18]^ Nevertheless, the potential involvement of *FAM110C* in DDR and its role in pancreatic cancer remain to be elucidated.

## Materials and methods

### Cells and different types of pancreatic tissues

Seven PDAC cell lines were involved in this study, including MIAPaCa-2, Panc3.11, Panc5.04, Panc10.05, SW1990, JF-305 and PATU-8988T. All cell lines were authenticated by STR profiling and routinely tested for mycoplasma contamination.

Different types of pancreatic tissue samples, including 284 cases of PDAC, 34 cases of IPMN and 15 cases of MCN, were obtained from the Chinese PLA General Hospital. The tumors were staged according to the 8th edition of the AJCC Cancer Staging Manual. None of the patients received chemotherapy before surgery. Sample collection followed the guidelines approved by the institutional review board (IRB number: 20090701–015).

### Cell treatment, RNA extraction, PCR

Cells were treated with 2 μM 5-aza-2’-deoxycytidine (5-aza, Sigma-Aldrich, # A3656, USA) for 96 h. Total RNA was extracted using TRIzol reagent (Invitrogen, #15596026, USA). Five micrograms of qualified RNA was used for cDNA synthesis following the manufacturer’s instruction (Thermo Scientific, #K1691, USA). The RT-PCR primer sequences for *FAM110C* and *GAPDH* (internal control) are listed in Supplementary Table 1.

### Preparation of DNA and sodium bisulfite treatment

A phenol-chloroform extraction assay was used to prepare DNA. Bisulfite treatment, methylation-specific PCR (MSP), and bisulfite sequencing (BSSQ) followed previous protocols.^[19,20]^ Normal lymphocyte DNA (NL) was used as a control for unmethylation, and *in vitro* methylated DNA (IVD) was used as a methylation control. The MSP and BSSQ primers for *FAM110C* are presented in Supplementary Table 1.

### Immunohistochemistry

Immunohistochemistry (IHC) was carried out following a previous description.^[20]^ The antibodies are listed in Supplementary Table 2.

### Construction of cell lines stably expressing *FAM110C*

The coding sequence of the human *FAM110C* (NM_001077710.3) expression vector was constructed using the pCDH-CMV-MCS-puro plasmid (Genewiz, AA27980-1/M548037, USA). FAM110C expressing or empty vectors with plasmids (pLP1, pLP2, and VSVG) were packaged using HEK293T cells with LipofectamineTM 3000 Reagent (Invitrogen, #L3000008, USA). Lentivirus-transfected PDAC cells were screened with puromycin (MCE, #HY-15695, USA) at concentrations of 1.5 μg/ mL (MIAPaCa-2) and 0.5 μg/mL (JF-305) for 3 days. Thereafter, monoclonal cells were selected by limited dilution in 96-well plates and validated by western blot.

### Construction of *FAM110C* knockout cell lines

The single guide RNA (sgRNA) sequences utilized in this study can be found in Supplementary Table 1. The sgRNAs targeting the first and second exons of *FAM 110C* were designed by the MIT CRISPR design tool (http:// crispr.mit.edu). The LentiCRISPR v2-gDNA plasmid was used to construct *FAM110C* knockout Panc10.05 cells. Monoclonal cells were selected with puromycin (2 μg/mL) following the above method.

### MTT and colony formation

PDAC cells were seeded into 96-well plates at a density of 2×10^3^ (MIAPaCa-2), 1.5×10^3^ (JF-305), and 1.5×10^3^ (Panc10.05) cells per well. An MTT assay was performed at 0, 24, 48, 72, and 96 h to determine cell viability (KeyGEN Biotech, # KGT5251, China). The results are shown as plotting curves, with the mean value ± standard deviation.

**Table 1.** The association betweenClinical factors and FAM110C methylation status inpancreaticcancer patients

	Methylation status			
Clinical parameter				
		Unmethylated	Methylated	
	NO. 284	*n* = 77 (27.18%)	*n* = 207 (72.89%)	*P* value
Gender				
Male	185	45	140	0.1485
Female	99	32	67	
Age (y)				
**≤**50	45	12	33	0.9415
>50	239	65	174	
Differentiation				
Well/Moderately	137	40	97	0.4456
Poorly	147	37	110	
TNM stage				
I/II	252	71	181	0.2586
III/IV	32	6	26	
Lymph node metastasis				
Negative	184	50	134	0.9749
Positive	100	27	73	
Tumor size (cm)				
**≤** 4 cm	217	67	150	0.0103*
>4 cm	67	10	57	
Tumor location				
Proximal	178	46	132	0.5327
Distal	106	31	75	
Smoking				
Yes	113	27	86	
No	171	50	121	0.3212
Alcohol consumption				
Yes	130	29	101	0.0942
No	154	48	106	

*P* values are obtained from χ^2^ test, *P < 0.05.

For colony formation, cells were seeded at a density of 300 (MIAPaCa-2), 300 (JF-305) and 500 (Panc10.05) cells per well in 6-well plates. The results were evaluated after a growth of 2 weeks.

### Cell Cycle and Apoptosis Analysis

*FAM 110C* silenced and re-expressed MIAPaCa-2 and JF-305 cells, as well as before and after *FAM110C* knockout Panc10.05 cells were synchronized to the G0/G1 phase by serum withdrawal for 12 h, followed by re-entry into the cell cycle by the addition of serum (10% FBS) for 36 h. Cells were fixed and stained with propidium iodide using a cell cycle detection kit (KeyGEN Biotech, #KGA512, China) in accordance with the instructions. For cell-cycle analysis, FACS Caliber (BD Biosciences, USA) and Modifit software (Verity Software House, USA) were employed. The apoptosis assay was performed following the instructions of the Annexin V-FITC/PI Apoptosis Detection Kit (KeyGEN Biotech, #KGA108, China).

### Transwell Assay

For cell migration evaluation, 6×10^4^ MIAPaCa-2, 2×10^4^ JF-305, and 4×10^4^ Panc10.05 cells were applied to the upper chamber (Corning, #3422, USA) for 30 h. In the invasion assay, 8×10^4^ MIAPaCa-2, 5×10^4^ JF-305, and 6×10^4^ Panc10.05 cells were placed in the upper chamber coated with matrigel (BD Biosciences, #354234, USA) for 36 h.

**Table 2.** Univariate and multivariate Coxregression analysis of prognosticfactors in PDAC patients(*n* = 186)

Clinical parameter	Univariate analysis		Multivariate analysis	
	HR (95%CI)	*P* value	HR (95%CI)	*P* value
Gender (male *vs*. female)	0.973 (0.649-1.460)	0.896		
Age (y) (**≤**50 *vs*. >50)	0.720 (0.394-1.316)	0.285		
Differentiation (high or middle *vs*. low differentiation)	0.696 (0.471-1.028)	0.069	0.727 (0.492, 1.075)	0.110
TNM stage (I/II *vs*. III/IV)	0.712 (0.417-1.216)	0.213		
Lymph node metastasis (negative *vs*. positive)	0.714 (0.485-1.052)	0.089	0.794 (0.536, 1.177)	0.250
Tumor size (cm) (<4 *vs*. 4) **≥**	0.822 (0.555-1.218)	0.329		
Tumor location (distal *vs*. proximal)	0.668 (0.443-1.008)	0.054	0.727 (0.479, 1.103)	0.134
*FAM110C* (unmethylation *vs*. methylation)	0.512 (0.313-0.837)	0.008^**^	0.544 (0.332, 0.893)	0.016^*^
Smoking (no *vs*. yes)	1.049 (0.707-1.556)	0.811		
Alcohol consumption (no *vs*. yes)	0.915 (0.621-1.349)	0.654		

HR: Hazard ratio; *P < 0.05; **P<0.01.

The procedures followed previous protocols.^[21]^

### Western blot and immunoprecipitation

Antibodies for western blot and immunoprecipitation (IP) are listed in Supplementary Table 2. A Rabbit IgG (Beyotime, #A7016, China) was employed as a negative control for IP. The procedures followed the manufacturer’s instructions (Thermo Scientific, #87788, USA; YEASEN, #36403ES08, China). Visible specific bands were resected for mass spectrometry analysis after SDS-PAGE electrophoresis and silver staining.

### SiRNA interference technique

SiRNAs against HMGB1 (siRNA#1, siRNA#2, and siRNA#3) and a negative control duplex (Scramble) were synthesized by JTSBIOCo. , Ltd (Wuhan, China) and transfected into the cells using RNAiMax (Invitrogen, #13778150, USA). The sequences of HMGB1-targeting siRNAs and the negative control duplex are listed in Supplementary Table 3. SiRNA#2 was determined to be the most efficient siRNA, and was applied for subsequent experiments.

### Xenograft mouse model

The animal experiment procedures were approved by the Animal Ethics Committee of the Chinese PLA General Hospital. BALB/c nude mice (4 weeks old) were purchased from SPF (Beijing, China) Biotechnology Co., Ltd. and housed under standard pathogen-free conditions. The nude mice were randomly divided into two groups, each consisting of five mice. FAM110C unexpressed and stably expressed MIAPaCa-2 cells (6×106) were used to build xenograft mice model. Tumor volume (V) was detected every 4 days following inoculation and calculated by the formula V = (length × width^2^) / 2.

### Half-inhibitory concentration analysis

Cells (MIAPaCa-2, JF-305, and Panc10.05) with or without *FAM110C* expression were seeded at a density of 3000 cells/well into 96-well plates. Cell viability was evaluated by MTT assay 48 h after treatment with gradient dilutions of ATR or CHK1 inhibitors (KeyGEN Biotech, #KGT5251, China). The absorbance was measured at a wavelength of 490 nm by a microplate reader (Thermo Multiskan MK3, USA). The value of the half-inhibitory concentration (IC_50_) was calculated using GraphPad Prism software (GraphPad Software Inc., USA).

### Statistical analysis

SPSS 22.0 software (IBM, USA) and GraphPad Prism 7.0 software (GraphPad Software Inc., USA) were employed in this study. The *chi-square* test was used to analyze the associations between *FAM110C* methylation status and clinicopathological factors. Kaplan-Meier survival curves were plotted with log-rank tests to compare overall survival (OS). Univariate and multivariate Cox regression analyses were used to evaluate prognostic factors for OS. Quantitative data was analyzed by the Student’s two-tailed *t* test. *P* < 0.05 was considered statistically significant.

## Results

### The expression of *FAM110C* is regulated by promoter region methylation in human PDAC

K450 microarray methylation and mRNA expression data from 178 cases of available PDAC were extracted from the UCSC Xena Browser (http://xena.ucsc.edu/). The expression of *FAM110C* was reduced in PDAC compared to noncancerous pancreatic tissue samples (*P* = 0.0000, Supplementary Figure 1A). The mRNA levels of *FAM110C* were inversely associated with methylation status in the CpG sites around transcription start site (TSS) (Supplementary Figure 1B), suggesting the possibility for methylation regulation of *FAM110C* gene expression.

**Figure 1:**
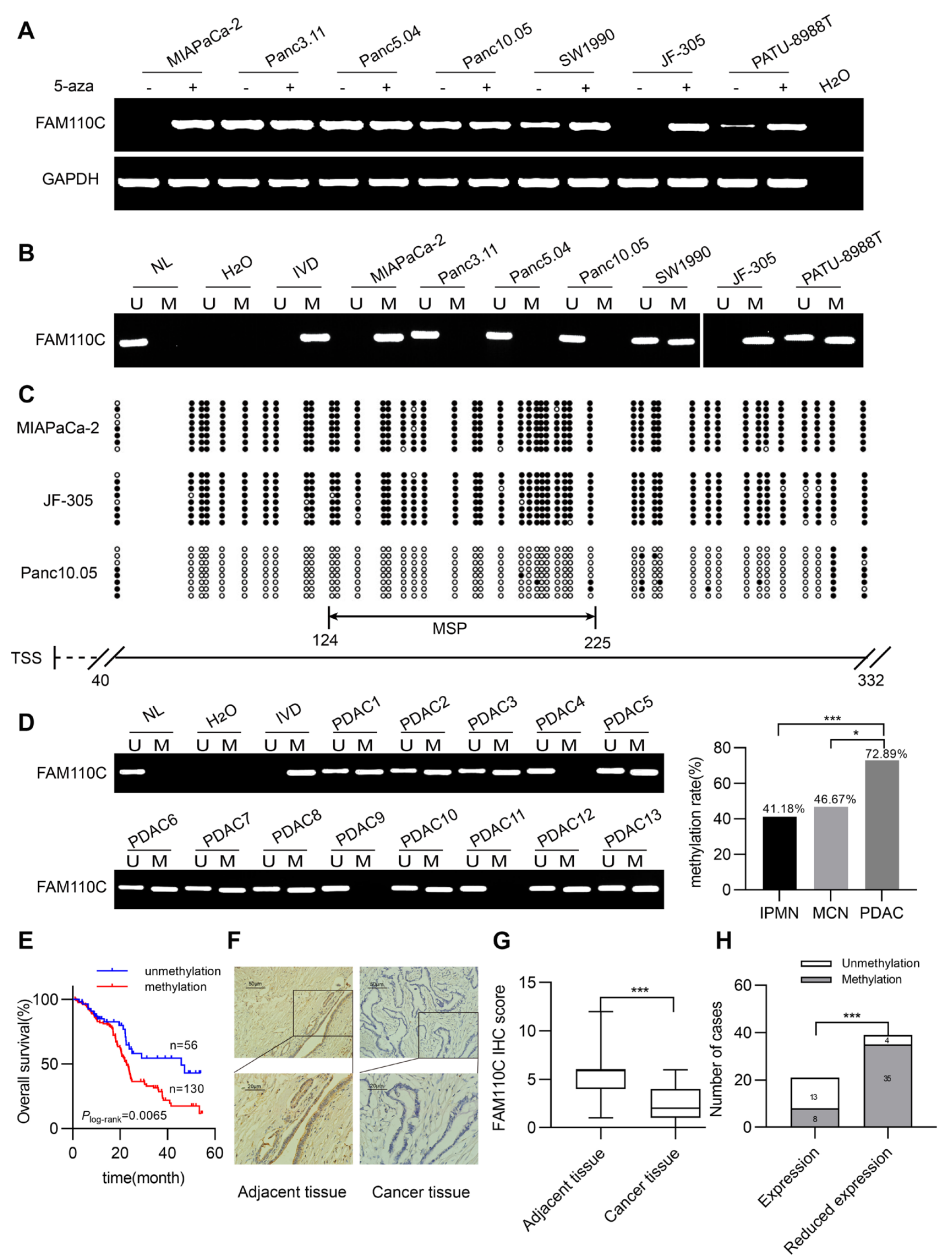
The expression and methylation status of *FAM110C* in pancreatic cancer cells and tissue samples. (A) MIAPaCa-2, Panc3.11, Panc5.04, Panc10.05, SW1990, JF-305, PATU-8988T are pancreatic cancer cells. H2O: negative control; GAPDH: internal control; 5-aza: 5-aza-2’-deoxycytidine;(-): absence of 5-aza;(+): presence of 5-aza. (B) MSP results present methylation status of *FAM110C* in pancreatic cancer cells. U: unmethylated alleles; M: methylated alleles; IVD: *in vitro* methylated DNA (methylation control); NL: normal lymphocytes DNA (unmethylation control); H2O: double distilled water. (C) BSSQ results of *FAM110C* in MIAPaCa-2, JF-305 and Panc10.05 cells. Double-headed arrow indicates the product size of MSP was 102 bp (from 124bp to 225bp) and bisulfite sequencing was conducted in a 293 bp region of the CpG island (from 40bp to 332bp) around the *FAM110C* transcription start site. Filled circles: methylated CpG sites; open circles: unmethylated CpG sites; TSS: transcription start site. (D) Representative MSP results of *FAM110C* in PDAC samples (*P < 0.05, ***P < 0.001). (E) The overall survival time was significantly shorter in methylated patients than in unmethylated individuals (*P* = 0.0065, Log-rank test). (F) Representative IHC results show *FAM110C* staining in pancreatic cancer tissue and adjacent tissue samples (top: 200×; bottom: 400×). (G) Box plots for *FAM110C* IHC score, horizontal lines represent the median score, vertical bars represent the range of score. ***P < 0.001. (H) Bar diagram indicates an inverse relationship between *FAM110C* expression levels and DNA methylation status. ***P < 0.001.

Then, the mRNA level of *FAM 110C* was examined by RT-PCR. It was not detected in MIAPaCa-2 and JF-305 cells, exhibited reduced expression in SW1990 and PATU-8988T cells, and exhibited higher expression levels in Panc3.11, Panc5.04 and Panc10.05 cells (Figure 1A). *FAM110C* was completely methylated in MIAPaCa-2 and JF-305 cells, partially methylated in SW1990 and PATU-8988T cells and unmethylated in Panc3.11, Panc5.04 and Panc10.05 cells (Figure 1B). The regulation of *FAM 110C* expression by methylation was further validated by 5-aza treatment (Figure 1A). MSP efficiency and methylation density were validated by BSSQ in MIAPaCa-2, JF-305 and Panc10.05 cells (Figure 1C).

Methylation of *FAM 110C* was detected in 41.18% (14/34) of IPMN, 46.67% (7/15) of MCN and 72.89% (207/284) of PDAC. The ratio of methylation is increased with the progression of carcinogenesis (*P* = 0.0001, *P* = 0.0389, Figure 1D). *FAM110C* methylation was significantly associated with tumor size (*P* = 0.0103), while no association was observed between *FAM 110C* methylation and gender, age, smoking, alcohol consumption, tumor differentiation, TNM staging, lymph node metastasis or tumor location (Table 1). For 186 cases of patients with available survival data, log rank testing was performed. *FAM110C* methylation was significantly associated with poor OS (*P* = 0.0065, Figure 1E). Then, univariate and multivariate Cox regression analyses were used to analyze the prognostic markers for survival. *FAM 110C* methylation is an independent prognostic marker for poor OS (*P* = 0.0159, Table 2).

The IHC assay was used for *FAM110C* expression detection in 60 available PDAC and matched adjacent tissue samples. The levels of *FAM110C* were higher in adjacent tissue than in PDAC tissue samples (*P* = 0.0000, Figure 1F& G). The staining was located in both the nucleus and cytoplasm (Figure 1F). Among the tumor samples, a reduced level of *FAM 110C* was associated with promoter region methylation (*P* = 0.0000, Figure 1H), indicating the epigenetic regulation of *FAM 110C* expression.

### *FAM110C* suppresses PDAC cell proliferation

**Figure 2:**
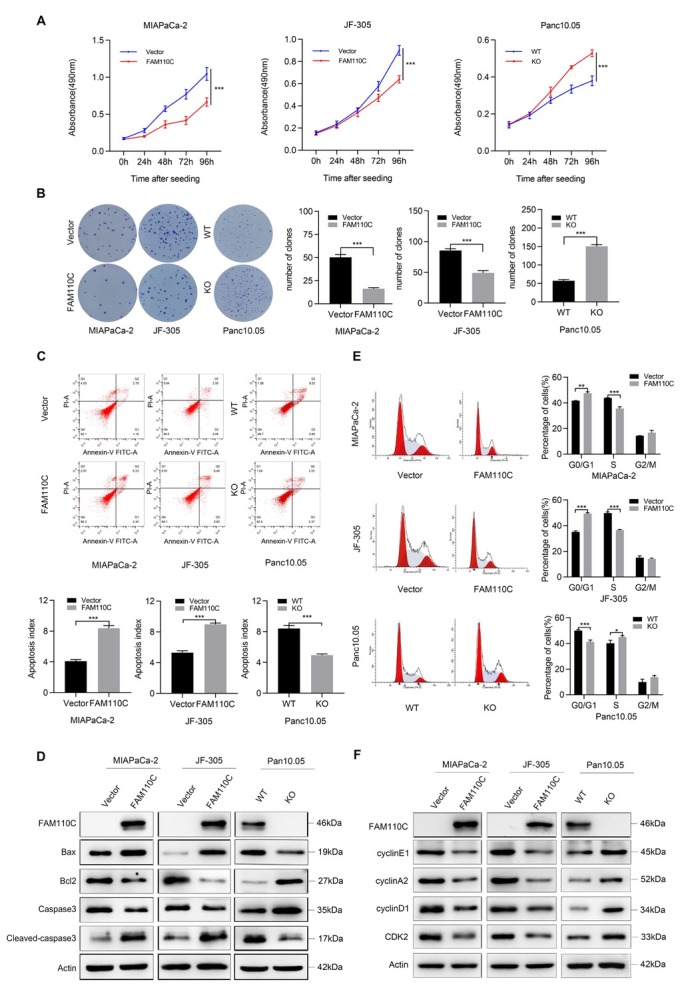
The roles of *FAM110C* play in cell proliferation, apoptosis and cell cycle. (A) OD value for *FAM110C* expressed and unexpressed MIAPaCa-2, JF-305 and Panc10.05 cells. (B) Representative colony formation results. (C) Apoptosis results of *FAM110C* expressed and unexpressed MIAPaCa-2, JF-305 and Panc10.05 cells. The bar diagram represents the percentage of apoptosis. (D) Western blot shows the effects of *FAM110C* on the expression levels of bax, bcl-2, caspase-3 and cleaved caspase-3. (E) Representative results of cell phase distribution. The bar diagram represents the percentage. (F) Western blot suggests the effects of *FAM110C* on expression levels of cyclin E1, cyclin A2, cyclinD1 and CDK2. Vector: control vector; *FAM110C*: *FAM110C* expressing vector; WT: wild type control; KO: *FAM110C* knockout. *P < 0.05; **P < 0.01; ***P < 0.001.

The OD values obtained by MTT assay were used for evaluating cell viability. The OD values were 1.045 ± 0.086 *vs*. 0.665 ± 0.056 and 0.900 ± 0.044 *vs*. 0.640 ± 0.033 before and after the re-expression of *FAM110C* in MIAPaCa-2 and JF-305 cells, respectively (Figure 2A). The OD value was decreased significantly by *FAM110C* (*P* = 0.0000, *P* = 0.0000). In Panc10.05 cells, the OD values were 0.380 ± 0.026 *vs*. 0.529 ± 0.019 before and after deletion of *FAM 110C* (*P* = 0.0000, Figure 2A). The above results indicate the inhibitory role of *FAM 110C* in cell proliferation.

The clone numbers were 50.3 ± 3.06 *vs*. 16.3 ± 1.16 and 85.7 ± 2.89 *vs*. 49.3 ± 3.51 in *FAM110C* unexpressed and re-expressed MIAPaCa-2 and JF-305 cells, respectively (*P* = 0.0001, *P* = 0.0002, Figure 2B). In *FAM110C* highly expressed and knockout Panc 10.05 cells, the clone numbers were 82.0 ± 5.10 *vs*. 128.7 ± 2.62 (*P* = 0.0000, Figure 2B). These results demonstrated that *FAM110C* inhibited PDAC cell colony formation.

### *FAM110C* induces cell apoptosis

The ratio of apoptotic cells was 4.09% ± 0.23% *vs*. 8.4% ± 0.35% and 5.31% ± 0.25% *vs*. 8.99% ± 0.17% in *FAM110C* unexpressed and re-expressed MIAPaCa-2 and JF-305 cells, respectively (*P* = 0.0001, *P* = 0.0000, Figure 2C). The ratio of apoptotic cells was 8.41% ± 0.42% *vs*. 4.96% ± 0.14% in Panc10.05 cells with high *FAM110C* expression and knockout (*P* = 0.0002, Figure 2C). These results suggested that *FAM110C* induces PDAC cell apoptosis. Restoration of *FAM110C* expression decreased caspase-3 and bcl-2 levels and increased cleaved caspase-3 and bax levels in MIAPaCa-2 and JF-305 cells, while knocking out *FAM110C* in Panc10.05 cells increased caspase-3 and bcl-2 levels and decreased cleaved caspase-3 and bax levels (Figure 2D), further suggesting the function of *FAM110C* in cell apoptosis.

### *FAM110C* induces G1/S phase arrest

In *FAM110C* silenced and re-expressed MIAPaCa-2 cells, the cell phase distributions were as follows: 41.76% ± 0.10% *vs*. 47.49% ± 1.17% for G0/G1 (*P*= 0.0011), 43.89% ± 0.19% *vs*. 35.66% ± 1.35% for S (*P* = 0.0005), and 14.35% ± 0.14% *vs*. 16.84% ± 1.76% for G2/M. For *FAM110C* unexpressed and re-expressed JF-305 cells, the distribution of cell phases was as follows: G0/G1 phase: 35.14% ± 0.81% *vs*. 49.34% ± 0.69% (*P* = 0.0000), S phase: 49.73% ± 0.94% *vs*. 36.59% ± 0.50% (*P* = 0.0000) and G2/M phase: 15.13% ± 1.29% *vs*. 14.07% ± 0.62%. *FAM110C* was deleted by the CRISPR CAS9 technique in highly expressed Panc10.05 cells, and cell phases were distributed as follows: G0/G1 phase: 50.03% ± 0.67% *vs*. 41.25% ± 1.42% ( *P* = 0.0006), S phase: 40.09% ± 2.35% *vs*. 45.07% ± 1.07% (*P* = 0.0289), and G2/M phase: 9.88% ± 2.27% *vs*. 13.69% ± 1.45% before and after *FAM110C* knockout. These results indicate that G1/S arrest is induced by *FAM110C* (Figure 2E). Decreased cyclin D1, cyclin A2, cyclin E1 and CDK2 levels were observed by restoration of *FAM110C* expression in MIAPaCa-2 and JF-305 cells, while increased cyclin D1, cyclin A2, cyclin E1 and CDK2 levels were observed in Panc10.05 cells by knocking out *FAM110C* (Figure 2F), further validating that *FAM110C* induces G1/S arrest.

### *FAM110C* suppresses cell migration and invasion

**Figure 3:**
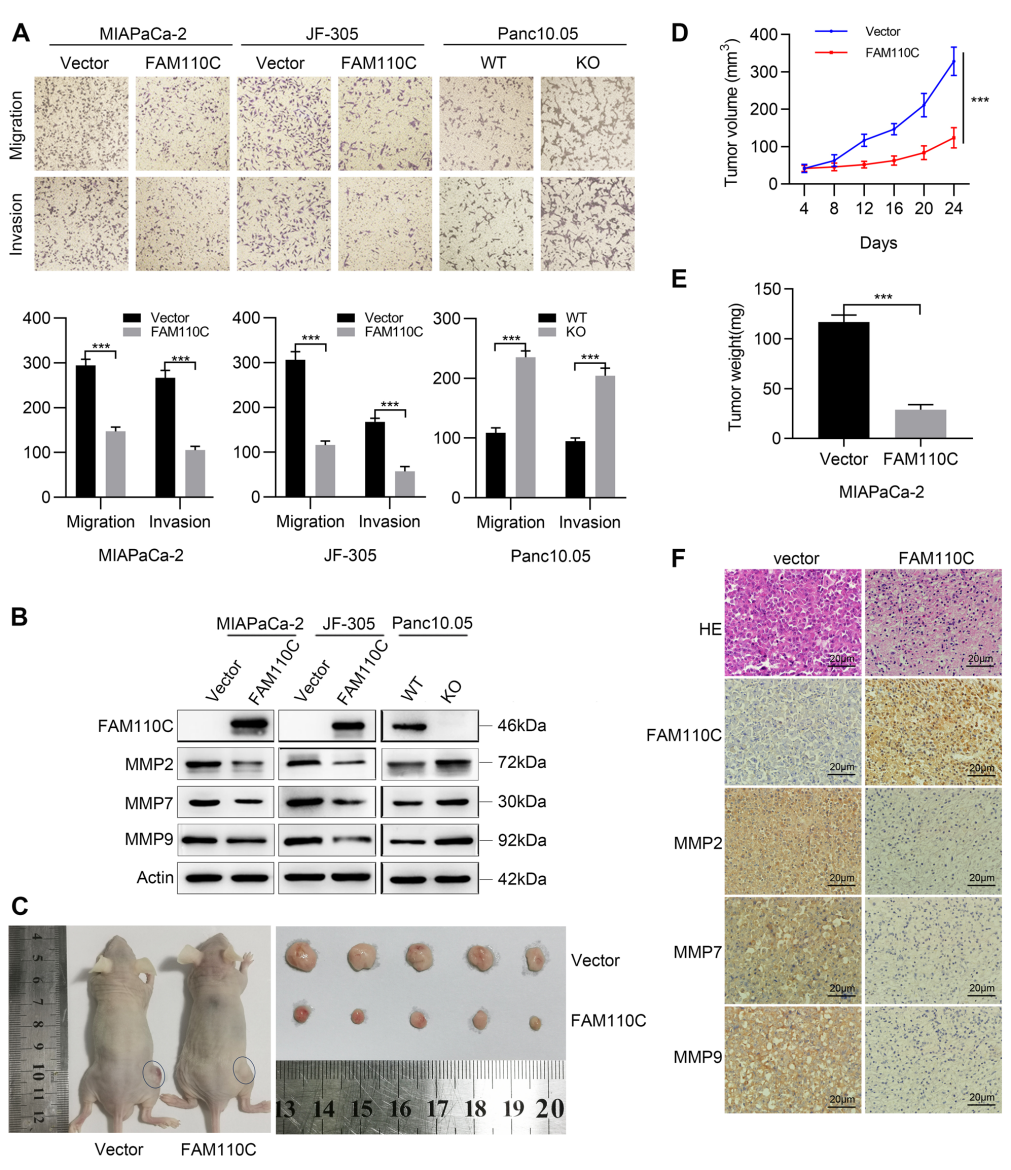
Effect of *FAM110C* on cell migration, invasion, and tumor xenograft model. (A) The migration and invasion results of *FAM110C* expressed and unexpressed MIAPaCa-2, JF-305 and Panc10.05 cells. The average number of migration and invasion cells was presented by the bar diagram. Vector: control vector; *FAM110C*: *FAM110C* expressing vector; WT: wild type control; KO: *FAM110C* knockout. ***P < 0.001. (B) Western blot shows the effects of *FAM110C* on the expression levels of MMP2, MMP7 and MMP9. (C) *FAM110C* unexpressed and re-expressed MIAPaCa-2 cells xenograft in nude mice. (D) Growth curves and (E) Tumor weight of *FAM110C* unexpressed and re-expressed MIAPaCa-2 cell xenografts (*n* = 5). ***P < 0.001. (F) The expression of MMP2, MMP7 and MMP9 in *FAM110C* unexpressed and re-expressed MIAPaCa-2 cell xenografts.

In *FAM110C* unexpressed and re-expressed MIAPaCa-2 and JF-305 cells, the number of migratory cells was 294.44 ± 1 3.91 *vs*. 147.11 ± 9.740 and 306.33 ± 18.33 *vs*. 115.89 ± 9.39, respectively (*P* = 0.0000, *P* = 0.0000, Figure 3A). The number of migratory cells before and after knockout of *FAM110C* in Panc10.05 cells was as follows: 108.67 ± 8.49 *vs*. 235.33 ± 10.74 (*P* = 0.0000, Figure 3A), further suggesting the inhibitory role of *FAM110C* in cell migration. For invasion analysis, in *FAM110C*-silenced and *FAM110C*-overexpressing MIAPaCa-2 and JF-305 cells, the invasive cells were 267.22 ± 16.08 *vs*. 105.33 ± 8.31 and 168.22 ± 7.97 *vs*. 57.56 ± 10.55, respectively (*P* = 0.0000, *P* = 0.0000, Figure 3A). The number of invasive cells was 94.67 ± 5.50 *vs*. 204.33 ± 12.70 in *FAM110C* highly expressed and knocking out Panc10.05 cells (*P* = 0.0000, Figure 3A). The above data demonstrates that *FAM110C* suppresses cell invasion. To further validate the influence of *FAM110C* on cell invasion and migration, MMP2, MMP7 and MMP9 were detected by western blot. Decreased MMP2, MMP7 and MMP9 were found in *FAM110C* re-expressed MIAPaCa-2 and JF-305 cells, and they were increased in *FAM110C* knockout Panc10.05 cells (Figure 3B), validating the results at the molecular level. The levels of MMP2, MMP7 and MMP9 were examined by IHC in *FAM110C* unexpressed and re-expressed MIAPaCa-2 cell xenografts. The levels of MMP2, MMP7 and MMP9 were decreased by re-expressing *FAM110C*, demonstrating the effect of *FAM110C* on invasion and migration *in vivo* (Figure 3F).

### The growth of PDAC cell xenografts was suppressed by *FAM110C*

In *FAM110C* defect and force-expressed MIAPaCa-2 cell xenografts, the tumor volume was 328.65 ± 38.38 *vs*. 123.91 ± 27.19 mm^3^ (*P* = 0.0000). Smaller tumor volumes were observed in xenografts re-expressing *FAM110C* (Figure 3C& D). The tumor weight was 116.68 ± 7.24 *vs*. 28.98 ± 4.97 mg in MIAPaCa-2 cell xenografts without and with forced expression of *FAM110C*. The tumor weight was significantly reduced by *FAM110C* (*P* = 0.0000, Figure 3E).

### *FAM110C* is involved in DNA damage repair by interacting with HMGB1

**Figure 4:**
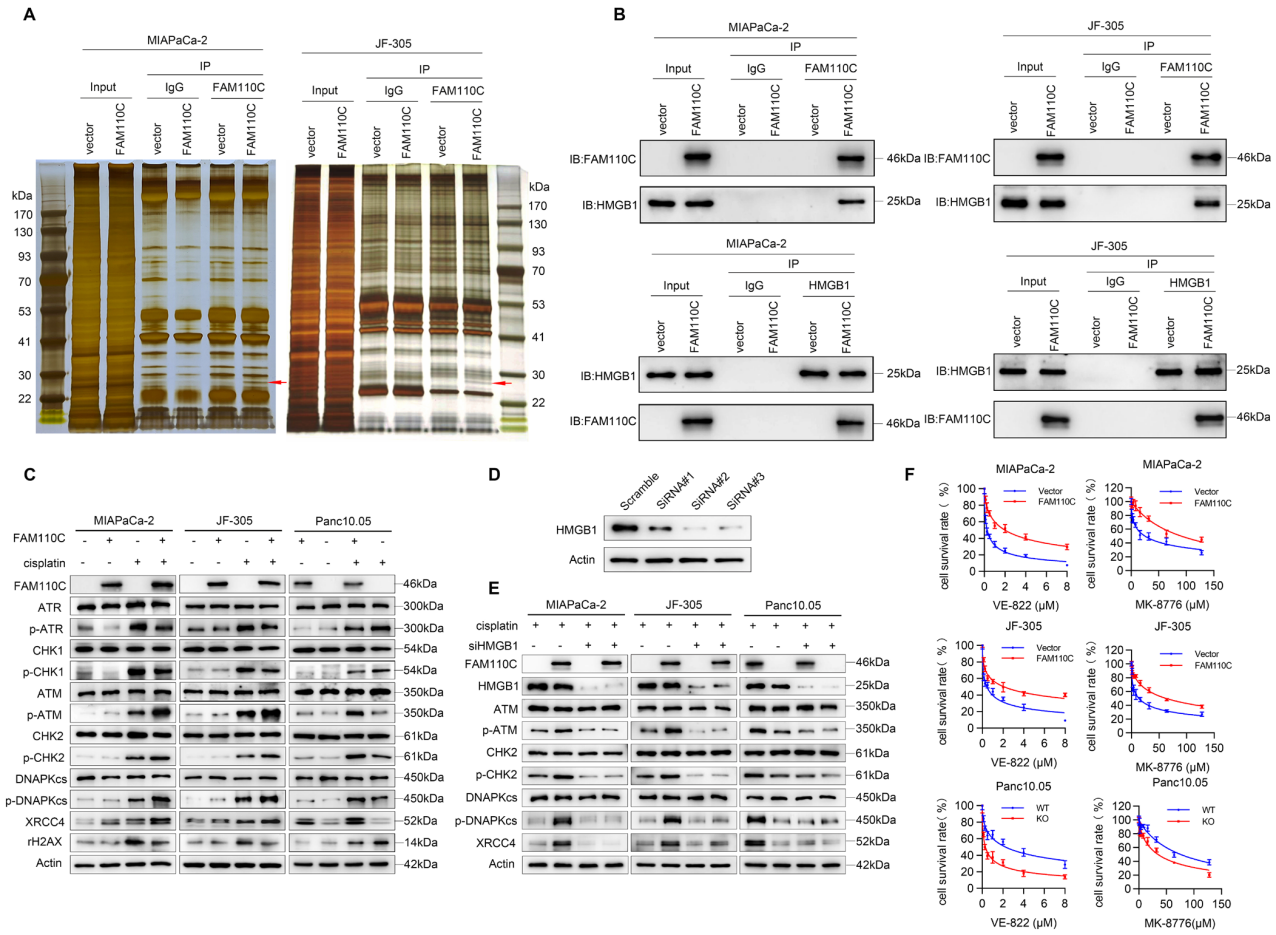
The role of *FAM110C* in DDR. (A) Co-IP assay and silver staining. Red arrow: specific band, which was subjected to mass spectrometry. IgG: negative control. *FAM110C* (-): without *FAM110C* expression *FAM110C* (+): *FAM110C* expression. (B) Validation of interaction between *FAM110C* and HMGB1. (C) cisplatin (-): without cisplatin treatment; cisplatin (+): cisplatin treatment. (D) Testing the efficiency of siRNAs for knocking down HMGB1. Scramble: siRNA negative control; siRNA#1, siRNA#2 and siRNA#3: siRNAs for HMGB1. (E) The effects of HMGB1 knockdown on ATM/CHK2 and NHEJ pathways. (F) Evaluation of IC50 for VE-822 and MK-8776 in PDAC cells.

To gain a deeper understanding of the roles of *FAM110C* in PDAC, an immunoprecipitation technique was employed. Figure 4A illustrates that when comparing PDAC cells with and without *FAM110C* expression, two extra bands were observed in *FAM110C* re-expressing MIAPaCa-2 and JF-305 cells. To identify the proteins present in these bands, mass spectrometry was utilized. As listed in Supplementary Table 4 and 5, the proteins that were pulled down by *FAM110C* were found to be similar in the two experiments. After excluding keratin, actin, and other cytoskeletal proteins, the majority of the proteins were related to apoptosis, DDR and stress-related signaling pathways. High mobility group box 1 (HMGB1) was observed to be present in both protein complexes derived from *FAM110C* re-expressed MIAPaCa-2 and JF-305 cell lysates. As shown in Supplementary Table 4 and 5, HMGB1 exhibited a higher score level, in addition to apoptosis-related proteins. HMGB1 was reported to be involved in DDR and stress-related signaling pathways.^[22, 23, 24, 25, 26]^ The interaction of *FAM110C* and HMGB1 was then validated by western blot and reciprocal co-IP assays (Figure 4B). Next, we focused on investigating the role of *FAM110C* through interacting with HMGB1 in PDAC cells under low dose cisplatin treatment, by comparing epigenetic silencing or deletion of *FAM110C* cells with *FAM110C* expressing cells.

The levels of phosphorylated ATR (p-ATR) and phosphorylated CHK1 (p-CHK1) were found to be elevated in *FAM110C* unexpresing MIAPaCa-2 and JF-305 cells compared to *FAM110C* re-expressing cells. Additionally, increased levels of p-ATR and p-CHK1 were observed after knockout of *FAM110C* in Panc 10.05 cells, indicating the inhibitory effect of *FAM110C* on the ATR/CHK1 pathway (Figure 4C). On the other hand, the levels of phosphorylated ATM (P-ATM) and phosphorylated CHK2 (p-CHK2) were increased after restoration of *FAM110C* expression in MIAPaCa-2 and JF-305 cells. Conversely, decreased levels of p-ATM and p-CHK2 were observed after knockout of *FAM110C* in Panc10.05 cells, demonstrating that *FAM110C* activated ATM signaling (Figure 4C). The effect of *FAM110C* on the non-homologous end joining (NHEJ) pathway was also assessed. In *FAM110C* expressing MIAPaCa-2, JF-305, and Panc10.05 cells, the levels of phosphorylated DNAPKcs (p-DNAPKcs) and XRCC4 were found to be higher than those in cells that did not express *FAM110C*, suggesting that *FAM110C* activated NHEJ signaling (Figure 4C).

To further validate the involvement of *FAM110C* in DDR through its interaction with HMGB1, siRNA was employed. The efficiency of siRNAs was tested, and siRNA#2 was found to be the most effective (Figure 4D). In *FAM110C* highly expressed MIAPaCa-2, JF-305 and Panc10.05 cells, the levels of p-ATM, p-CHK2, p-DNAPKcs and XRCC4 were reduced by knocking down HMGB1, further demonstrating that the effect of *FAM110C* on DDR is mediated through its interaction with HMGB1 (Figure 4E).

### Loss of *FAM110C* expression sensitizes pancreatic cancer cells to VE-822 and MK-8776

As *FAM110C* is involved in NHEJ and ATM signaling, and ATR/CHK1 signaling is the compensation pathway, we explored the sensitivity of PDAC cells to VE-822 (an ATR inhibitor) and MK-8776 (a CHK1 inhibitor), with or without *FAM110C* expression. The IC_50_ of VE-822 was 0.281 ± 0.074 μM *vs*. 2.011 ± 0.226 μM and 0.441 ± 0.071 μM *vs*. 2.096 ± 0.184 μM in *FAM110C* unexpressed and re-expressed MIAPaCa-2 and JF-305 cells under treatment with cisplatin, respectively. The IC_50_ of VE- 822 was reduced significantly in *FAM110C*-silenced cells (*P* = 0.0000, *P* = 0.0000, Figure 4F). The IC_50_ of VE-822 was 2.268 ± 0.469 μM *vs*. 0.337 ± 0.128 μM in Panc10.05 cells before and after knockout of *FAM110C*. The IC_50_ was significantly reduced after knockout of *FAM110C* (*P* = 0.0000, Figure 4F). These results indicate that loss of *FAM110C* expression sensitized PDAC cells to the ATR inhibitor.

The IC_50_ of MK-8776 was 26.330 ± 8.128 μM *vs*. 93.682 ± 11.243 μM and 13.442 ± 1.632 μM *vs*. 63.373 ± 12.309 μM in *FAM110C* unexpressed and re-expressed MIAPaCa-2 and JF-305 cells under treatment with cisplatin, respectively (*P* = 0.0000, *P* = 0.0000, Figure 4F). The ICof MK-8776 was 76.623 ± 9.618 μM *vs*. 34.748 ± 4.387 μM in Panc10.05 cells before and after knockout of *FAM110C* under cisplatin treatment (*P* = 0.0000, Figure 4F). These results demonstrated that loss of *FAM110C* expression sensitized pancreatic cancer cells to the CHK1 inhibitor.

## Discussion

Abnormal epigenetic alterations have been reported in various cancers, and DNA methylation is regarded as a potential cancer detection, prediction, prognosis and chemo-radio therapeutic marker.^[27, 28, 29]^ The nature of epigenetic changes is reversible, making it an attractive therapeutic target. Gene expression is regulated by epigenetic machinery, including writers (responsible for adding modifications to DNA or histones, such as DNA methyltransferase), readers (recognition of modifications and recruitment of effector proteins, such as methyl-binding domain proteins) and erasers (enzymes to remove chemical modifications, such as histone demethylase).^[12,30]^ Many drugs have been developed to target epigenetic machinery.^[28,31]^ Two demethylating agents, decitabine and azacytidine, were approved by the FDA in hematological malignancies and myelodysplastic syndromes.^[31,32]^ Significant toxicity was found with high-dose treatment in solid tumors, without improving overall survival.^[30,33]^ Accumulating evidence demonstrates that the efficacy of mono epi-drug therapy is very limited, suggesting that specific restoration of epigenetically silenced gene expression is challenged markedly by epi-drugs. Clinical trials involving the combination of chemotherapy, radiotherapy, or immunotherapy with epi-drug therapy are currently underway.^[34, 35, 36]^ An epigenetic-based therapeutic strategy employing synthetic lethality may precisely target cancer cells with aberrant epigenetic changes, without hurting normal cells.^[11,33]^ The concept of synthetic lethality stems from the study of fruit flies, a genetic model. A lethal outcome is observed when both specific genes are mutated, whereas individual mutations of either gene alone do not affect viability.^[37]^ This principle was applied to cancer therapy with PARP inhibitors in BRAC1/2 mutated cells.^[38,39]^ The rationale was extended to “BRCAness” for other DDR gene mutants.^[40,41]^ Beyond “BRCAness”, epigenetic silencing of DDR-related or cell fate-related genes is also suitable for synthetic lethal therapeutic strategies.^[21,42]^ It is desirable to look for novel DDR-related or cell fate-related genes, that are regulated by epigenetics and have aberrant epigenetic changes in tumors to broaden the scope of therapeutic targets.

*FAM110A* and *FAM110B* were reported to play important roles in pancreatic, lung, and prostate cancers.^[16,43,44]^ However, the role of *FAM110C* in cancer remains unclear. It is important to understand the epigenetic regulation and mechanism of *FAM110C* in pancreatic cancer to develop novel treatment strategies. Our results demonstrated that *FAM110C* was frequently methylated in PDAC, with an accumulating tendency during carcinogenesis. *FAM110C* methylation was significantly associated with tumor size. These results indicate that methylation of *FAM110C* may serve as a potential early PDAC detection marker. The log rank test was conducted on 186 cases of patients with available survival data, revealing a significant association between *FAM110C* methylation and poor OS. Subsequently, both univariate and multivariate Cox regression analyses were employed, confirming that *FAM110C* methylation is an independent prognostic marker for poor OS.

*FAM110C* suppressed PDAC cell proliferation, migration and invasion, and induced apoptosis and G1/S arrest. *FAM110C* suppressed PDAC cell xenograft growth in mice, implying its potential as a novel tumor suppressor in PDAC. To gain further insights into the mechanism of *FAM110C* in PDAC, an IP assay and mass spectrometry analysis were applied. The interaction of FAM110C and HMGB1 was discovered, and confirmed through western blot and reverse immunoprecipitation. HMGB1 is reported to be involved in the PI3K/AKT, NF-kB and JAK/STAT signaling pathways to regulate DNA replication and gene transcription.^[25,26]^ HMGB1 was also revealed to be involved in different DDR signaling pathways, including mismatch repair (MMR), base excision repair (BER), nucleotide excision (NER), and NHEJ.^[25,45, 46, 47, 48]^ Both the PI3K/AKT and NF-κB signaling pathways were reported to promote DDR and are involved in inflammation.^[11,49, 50, 51, 52, 53]^ Under the inflammatory environment, HMGB1 may promote carcinogenesis, especially during hepatocarcinogenesis.^[26,54]^ Therefore, HMGB1 may play conflicting roles in various cancers under different environments, potentially exerting both antitumor and protumor effects. HMGB1 was recently found to be a damage-associated molecule in dying cancer cells that enhances immunogenic cell death.^[55]^ Under DNA-damaging agent treatment in various malignant tumors, HMGB1 has been reported to primarily regulate the DNA damage response checkpoint and cell survival.^[22,56,57]^ Notably, chemotherapy agents predominantly induce DNA double strand breaks, which are repaired through the classical pathways of homologous recombination repair (HR) and NHEJ. HR is composed of the ATR/CHK1 and ATM/ CHK2 signaling pathways.^[58]^ Subsequently, an investigation was conducted to examine the role of *FAM110C* in DDR. These findings indicate that *FAM110C* activates the NHEJ and ATM/CHK2 signaling pathways while inhibiting the ATR/CHK1 pathway in PDAC cells.

**Figure 5:**
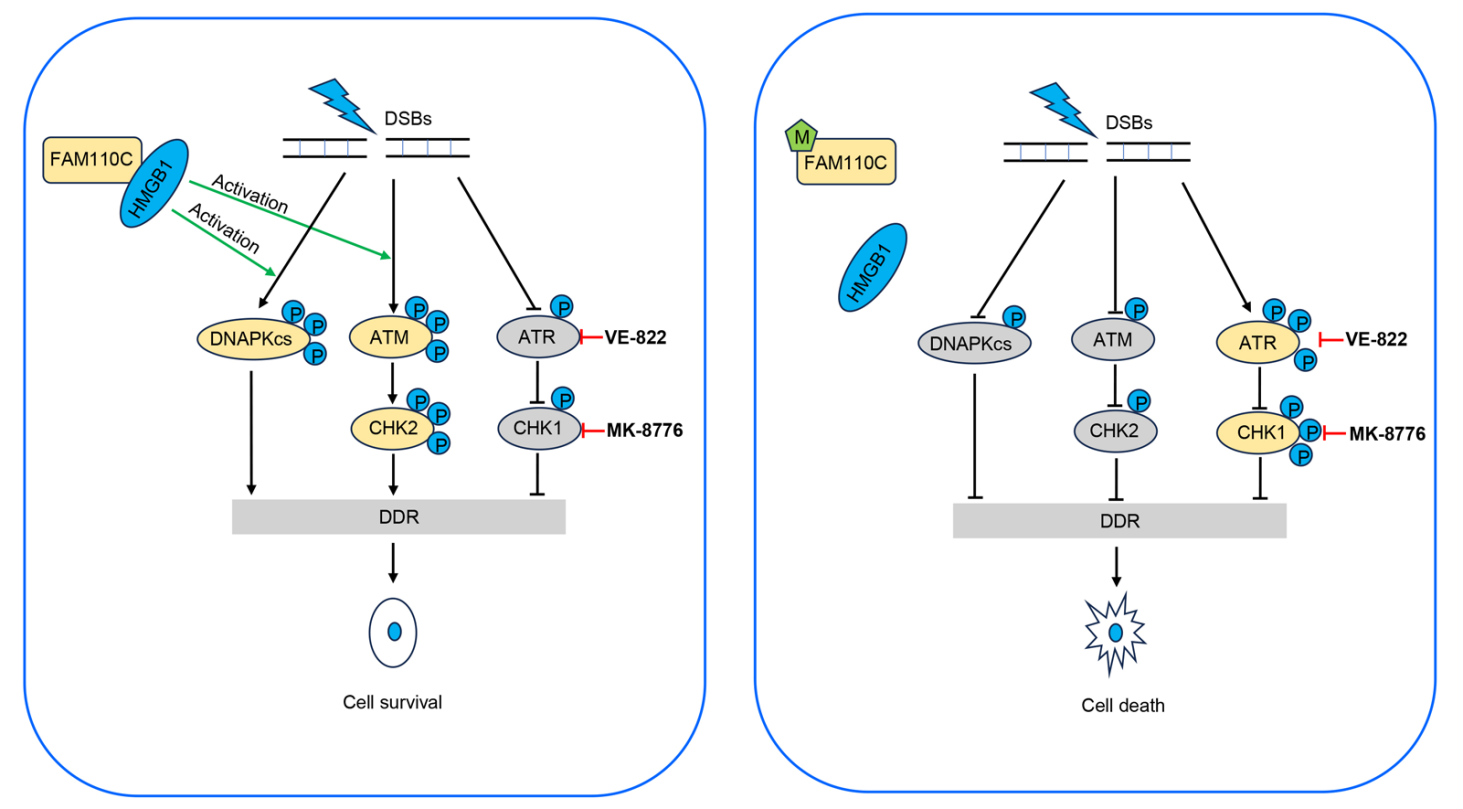
The schematic illustration of synthetic lethality between epigenetic silencing of FAM110C and ATR/CHK1 inhibitors. A working model for synthetic lethality of FAM110C methylation and ATR/CHK1 inhibitor in PDAC cells. DSBs: double-strand breaks; M: DNA methylation; P: phosphorylation; DDR: DNA damage repair.

Epigenetic-based synthetic lethality emerges as a novel strategy for cancer therapy. Consequently, an exploration was undertaken to assess the synthetic lethal effects between ATR/CHK1 inhibitors and the loss of *FAM110C* expression. ATR and CHK1 inhibitors have shown promise in treating PARP inhibitor-resistant PDAC, and ongoing clinical trials are evaluating the combination of ATR/CHK1 inhibitors with other therapeutics.^[59, 60, 61]^ Our findings indicated that the epigenetic silencing or deletion of *FAM110C* sensitized PDAC cells to ATR and CHK1 inhibitors when exposed to low doses of cisplatin (Figure 5). It is noteworthy that epigenetic abnormalities are more frequently observed in DDR and cell fate signaling pathways in various types of cancer.^[10]^ Therefore, conducting further investigations on *FAM110C* may lead to the development of more effective therapeutic strategies for PDAC.

In conclusion, *FAM110C* acts as a potential tumor suppressor in PDAC. *FAM110C* methylation is a potential diagnostic and prognostic marker for PDAC. Epigenetic silencing of *FAM110C* sensitized pancreatic cancer cells to ATR/CHK1 inhibitors.

## Supplementary Material

Supplementary Material
