# Supplementary material for: Methylation of FAM110C is a synthetic lethal marker for ATR/CHK1 inhibitors in pancreatic cancer
Source: J Transl Int Med. 2024 Jul 27;12(3):274–87. doi: 10.2478/jtim-2023-0128 (PMC11284899; doi:10.2478/jtim-2023-0128)
Supplement: Supplementary file 1 — Supplementary Material [file jtim-2023-0128_sm.pdf]

Supplementary Materials

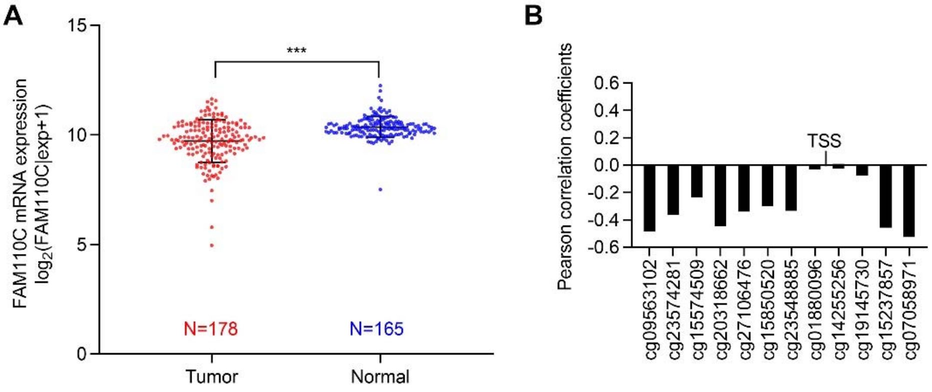

Supplementary figure 1 The expression and methylation status of *FAM110C* in TCGA and GTEx database. (A)The expression levels of *FAM110C* in pancreatic cancer tissue and normal tissue samples. ***P < 0.001. (B) The relationship between *FAM110C* expression and methylation at CpG sites in the promoter region in 178 cases of pancreatic cancer samples.

Supplementary table 1 The list of primer sequences.

Primer Names	Primer Sequences
FAM110C RT-PCR primer	5′-ACTGTGTGACCCTCAAGGTGC-3′(F)
	5′-TCATCGGGAAGGTTTGCTTCCTC-3′(R)
GAPDH RT-PCR primer	5′-GACCACAGTCCA TGCCATCAC -3′(F)
	5′-GTCCACCACCCTGTTGCTGTA-3′ (R)
FAM110C-MSP primer	5′- GATGCGCGTTTTGGCGGTTTTGAGC -3′ (MF)
	5′- CCTCTCCACTACGCTCCTACGCG -3′(MR)
	5′- GAAGATGTGTGTTTTGGTGGTTTTGAGT -3′ (UF)
	5′- CAACCTCTCCACTACACTCCTACACA -3′(UR)
FAM110C-BSSQ primer	5′- GAATYGGTTTTGGGATTGATTGG -3′(F)
	5-TCCCCRAACACTTAATCRCCC-3′(R)
FAM110C-CDS primer	5′- GCTCTAGAATGCGCGCCCTGGCGGCCCTGA-3′(F)
	5′- CGGGATCCTCATCGGGAAGGTTTGCTTCCTCGGG -3′(R)
sgRNA1	5′-CACCGCGGATCGCGCCAAGTATGTG -3′(F)
	5′-AAACCACATACTTGGCGCGATCCGC-3′(R)
sgRNA2	5′-CACCGATGCGAATTCGTGCGAGGAT -3′(F)
	5′-AAACATCCTCGCACGAATTCGCATC-3′(R)

F: forward; R: reverse; MF: methylated forward primer; MR: methylated reverse primer; UF: unmethylated forward primer; UR: unmethylated reverse primer.

Supplementary table 2 The list of antibodies.

Gene name	Manufacturer	Cat No.	Antigenicity	Usage
FAM110C	Novus Biologicals	NBP2-84878	Rabbit	IP 2μg
				WB 1:500
FAM110C	Invitrogen	PA5-57542	Rabbit	IHC 1:500
HMGB1	Proteintech	HX15100	Rabbit	IP 5μg
cyclinE1	Proteintech	11554-1-AP	Rabbit	WB 1:1000
cyclinA2	Proteintech	18202-1-AP	Rabbit	WB 1:10000
cyclinD1	Proteintech	60186-1-Ig	Mouse	WB 1:10000
CDK2	Proteintech	10122-1-AP	Rabbit	WB 1:10000
MMP2	Proteintech	66366-1-Ig	Mouse	WB 1:1000
				IHC 1:300
MMP7	ZENBIO	820096	Rabbit	WB 1:500
				IHC 1:300
MMP9	Proteintech	10375-2-AP	Rabbit	WB 1:1000
				IHC 1:300
Caspase3	Proteintech	19677-1-AP	Rabbit	WB 1:500
Cleaved-caspase3	Proteintech	19677-1-AP	Rabbit	WB 1:500
Bcl-2	Proteintech	12789-1-AP	Rabbit	WB 1:2000
Bax	Proteintech	50599-2-Ig	Rabbit	WB 1:2000
ATM	huaxingbio	HX12561	Rabbit	WB 1:1000
p-ATM(Ser1981)	huaxingbio	HX8919	Rabbit	WB 1:1000
CHK2	ZENBIO	R23921	Rabbit	WB 1:1000
p-CHK2(Thr68)	ZENBIO	340766	Rabbit	WB 1:1000
ATR	Cell signaling	2790S	Rabbit	WB 1:1000
p-ATR(Ser428)	Cell signaling	2853S	Rabbit	WB 1:1000
CHK1	ZENBIO	380200	Rabbit	WB 1:1000
p-CHK1(Ser345)	Gentex	GTX100065	Rabbit	WB 1:1000
DNA PKcs	ZENBIO	200618-6D1	Mouse	WB 1:1000
p-DNA PKcs (Ser2056)	ZENBIO	380800	Rabbit	WB 1:1000
XRCC4	huaxingbio	HX19688	Mouse	WB 1:1000
γ-H2AX (Ser139)	Cell Signaling	9718	Rabbit	WB 1:1000
β-actin	Proteintech	66009-1-Ig	Mouse	WB 1:50000

Supplementary table 3 The siRNA sequences for *HMGB1*.

Primer Names	Primer Sequences
Scramble	5'-UUCUCCGAACGUGUCACGUTT -3'(F)
	5'-ACGUGACACGUUCGGAGAATT-3'(R)
siRNA#1	5'-GGGAGGAGCAUAAGAAGAATT -3'(F)
	5'-UUCUUCUUAUGCUCCUCCCTT-3'(R)
siRNA#2	5'-GGACAAGGCCCGUUAUGAATT -3'(F)
	5'-UUCAUAACGGGCCUUGUCCTT-3'(R)
siRNA#3	5'-CCUCUUCUGCUCUGAGUAUTT -3'(F)
	5'-AUACUCAGAGCAGAAGAGGTT-3'(R)

F: forward; R: reverse.

Supplementary table 4 The list of proteins (score >3) from the extra band in FAM110C re-expressed MIAPaCa-2 cell.

Accession	Description	Score	related function / signal pathways
P27348	14-3-3 protein theta OS=Homo sapiens OX=9606 GN=YWHAQ PE=1 SV=1 - [1433T_HUMAN]	146.33	apoptosis
P63103	14-3-3 protein zeta/delta OS=Bos taurus OX=9913 GN=YWHAZ PE=1 SV=1 - [1433Z_BOVIN]	110.82	apoptosis
P31946-2	Isoform Short of 14-3-3 protein beta/alpha OS=Homo sapiens OX=9606 GN=YWHAB - [1433B_HUMAN]	85.55	apoptosis
P67936	Tropomyosin alpha-4 chain OS=Homo sapiens OX=9606 GN=TPM4 PE=1 SV=3 - [TPM4_HUMAN]	55.10	NF-κB, stress
P06753-3	Isoform 3 of Tropomyosin alpha-3 chain OS=Homo sapiens OX=9606 GN=TPM3 - [TPM3_HUMAN]	38.88	EMT
P07477	Trypsin-1 OS=Homo sapiens OX=9606 GN=PRSS1 PE=1 SV=1 - [TRY1_HUMAN]	37.74	ERK, apoptosis
P61981	14-3-3 protein gamma OS=Homo sapiens OX=9606 GN=YWHAG PE=1 SV=2 - [1433G_HUMAN]	34.86	apoptosis
P62701	40S ribosomal protein S4, X isoform OS=Homo sapiens OX=9606 GN=RPS4X PE=1 SV=2 - [RS4X_HUMAN]	30.40	MTOR, stress
P09493-5	Isoform 5 of Tropomyosin alpha-1 chain OS=Homo sapiens OX=9606 GN=TPM1 - [TPM1_HUMAN]	27.18	strss, cell fate, DNA damage repair
Q04917	14-3-3 protein eta OS=Homo sapiens OX=9606 GN=YWHAH PE=1 SV=4 - [1433F_HUMAN]	24.76	apoptosis
P09429	**High mobility group protein B1 OS=Homo sapiens OX=9606 GN=HMGB1 PE=1 SV=3 - [HMGB1_HUMAN]**	**20.70**	**DNA damage repair, PI3K/AKT, JAK/STAT and NF-κB**
P09493-10	Isoform 10 of Tropomyosin alpha-1 chain OS=Homo sapiens OX=9606 GN=TPM1 - [TPM1_HUMAN]	16.59	strss, cell fate, DNA damage repair
P31947-2	Isoform 2 of 14-3-3 protein sigma OS=Homo sapiens OX=9606 GN=SFN - [1433S_HUMAN]	14.90	apoptosis
Q86YZ3	Hornerin OS=Homo sapiens OX=9606 GN=HRNR PE=1 SV=2 - [HORN_HUMAN]	14.59	AKT, immune
Q8NC51-4	Isoform 4 of Plasminogen activator inhibitor 1 RNA-binding protein OS=Homo sapiens OX=9606 GN=SERBP1 - [PAIRB_HUMAN]	10.91	stress, apoptosis, metabolism
P35579-2	Isoform 2 of Myosin-9 OS=Homo sapiens OX=9606 GN=MYH9 - [MYH9_HUMAN]	10.06	stress, β-catenin, NOTCH, PI3K/AKT
P0CG48	Polyubiquitin-C OS=Homo sapiens OX=9606 GN=UBC PE=1 SV=3 - [UBC_HUMAN]	9.60	stress, ubiquitin
P18124	60S ribosomal protein L7 OS=Homo sapiens OX=9606 GN=RPL7 PE=1 SV=1 - [RL7_HUMAN]	9.55	stress, ferroptosis
P48507	Glutamate--cysteine ligase regulatory subunit OS=Homo sapiens OX=9606 GN=GCLM PE=1 SV=1 - [GSH0_HUMAN]	9.39	ferroptosis, metabolism, ERK/Nrf2, NF-κB/Nrf2
P47756-2	Isoform 2 of F-actin-capping protein subunit beta OS=Homo sapiens OX=9606 GN=CAPZB - [CAPZB_HUMAN]	8.58	cell fate
P26373	60S ribosomal protein L13 OS=Homo sapiens OX=9606 GN=RPL13 PE=1 SV=4 - [RL13_HUMAN]	6.89	NF-κB
Q9Y5M8	Signal recognition particle receptor subunit beta OS=Homo sapiens OX=9606 GN=SRPRB PE=1 SV=3 - [SRPRB_HUMAN]	5.89	NF-κB, stress
O60762	Dolichol-phosphate mannosyltransferase subunit 1 OS=Homo sapiens OX=9606 GN=DPM1 PE=1 SV=1 [DPM1_HUMAN]	4.91	stress, metabolism
P81605	Dermcidin OS=Homo sapiens OX=9606 GN=DCD PE=1 SV=2 - [DCD_HUMAN]	4.91	stress, Wnt
P35030-5	Isoform 5 of Trypsin-3 OS=Homo sapiens OX=9606 GN=PRSS3 - [TRY3_HUMAN]	4.04	MEK/ERK, chemoresisitance
P62917	60S ribosomal protein L8 OS=Homo sapiens OX=9606 GN=RPL8 PE=1 SV=2 - [RL8_HUMAN]	3.96	MTOR, ferroptosis
P04424-2	Isoform 2 of Argininosuccinate lyase OS=Homo sapiens OX=9606 GN=ASL - [ARLY_HUMAN]	3.79	metabolism
P05141	ADP/ATP translocase 2 OS=Homo sapiens OX=9606 GN=SLC25A5 PE=1 SV=7 - [ADT2_HUMAN]	3.43	PI3K/AKT, ERK, metabolism
P60660	Myosin light polypeptide 6 OS=Homo sapiens OX=9606 GN=MYL6 PE=1 SV=2 - [MYL6_HUMAN]	3.34	chemoresistant, cAMP Pathway

Supplementary table 5 The list of proteins (score >3) from the extra band in *FAM110C* re-expressed JF-305 cell.

Accession	Description	Score	related function / signal pathways
P15924	Desmoplakin OS=Homo sapiens OX=9606 GN=DSP PE=1 SV=3	40.328	p53, Wnt, EMT
P27348	14-3-3 protein theta OS=Homo sapiens OX=9606 GN=YWHAQ PE=1 SV=1	39.197	apoptosis
P63104	14-3-3 protein zeta/delta OS=Homo sapiens OX=9606 GN=YWHAZ PE=1 SV=1	36.903	apoptosis
P31946	14-3-3 protein beta/alpha OS=Homo sapiens OX=9606 GN=YWHAB PE=1 SV=3	31.732	apoptosis
P14923	Junction plakoglobin OS=Homo sapiens OX=9606 GN=JUP PE=1 SV=3	24.367	EMT, PI3K/AKT
P61981	14-3-3 protein gamma OS=Homo sapiens OX=9606 GN=YWHAG PE=1 SV=2	24.266	apoptosis
P62701	40S ribosomal protein S4, X isoform OS=Homo sapiens OX=9606 GN=RPS4X PE=1 SV=2	22.137	MTOR, stress
P26373	60S ribosomal protein L13 OS=Homo sapiens OX=9606 GN=RPL13 PE=1 SV=4	21.304	NF-κB
P06733	Alpha-enolase OS=Homo sapiens OX=9606 GN=ENO1 PE=1 SV=2	20.556	DNA damage repair
P67936	Tropomyosin alpha-4 chain OS=Homo sapiens OX=9606 GN=TPM4 PE=1 SV=3	20.49	NF-κB, stress
Q02413	Desmoglein-1 OS=Homo sapiens OX=9606 GN=DSG1 PE=1 SV=2	18.012	stress
P09493	Tropomyosin alpha-1 chain OS=Homo sapiens OX=9606 GN=TPM1 PE=1 SV=2	16.822	strss, cell fate, DNA damage repair
P23396	40S ribosomal protein S3 OS=Homo sapiens OX=9606 GN=RPS3 PE=1 SV=2	16.208	NF-κB, DNA damage repair
Q04917	14-3-3 protein eta OS=Homo sapiens OX=9606 GN=YWHAH PE=1 SV=4	14.995	apoptosis
P14618	Pyruvate kinase PKM OS=Homo sapiens OX=9606 GN=PKM PE=1 SV=4	13.689	metabolism
P68104	Elongation factor 1-alpha 1 OS=Homo sapiens OX=9606 GN=EEF1A1 PE=1 SV=1	13.049	AKT, apoptosis
O60762	Dolichol-phosphate mannosyltransferase subunit 1 OS=Homo sapiens OX=9606 GN=DPM1 PE=1 SV=1	12.902	stress, cell fate
P18669	Phosphoglycerate mutase 1 OS=Homo sapiens OX=9606 GN=PGAM1 PE=1 SV=2	12.685	ferroptosis, NF-κB,metabolism
P10809	60 kDa heat shock protein, mitochondrial OS=Homo sapiens OX=9606 GN=HSPD1 PE=1 SV=2	11.987	stress
P31947	14-3-3 protein sigma OS=Homo sapiens OX=9606 GN=SFN PE=1 SV=1	11.542	apoptosis
P07900	Heat shock protein HSP 90-alpha OS=Homo sapiens OX=9606 GN=HSP90AA1 PE=1 SV=5	10.43	stress
P81605	Dermcidin OS=Homo sapiens OX=9606 GN=DCD PE=1 SV=2	10.232	stress, Wnt
Q5D862	Filaggrin-2 OS=Homo sapiens OX=9606 GN=FLG2 PE=1 SV=1	10.093	JAK/STAT
O60814	Histone H2B type 1-K OS=Homo sapiens OX=9606 GN=H2BC12 PE=1 SV=3	9.766	histone
P08238	Heat shock protein HSP 90-beta OS=Homo sapiens OX=9606 GN=HSP90AB1 PE=1 SV=4	9.479	stress
Q9HB71	Calcyclin-binding protein OS=Homo sapiens OX=9606 GN=CACYBP PE=1 SV=2	9.293	p53, MAPK , apoptosis, Wnt
P35232	Prohibitin 1 OS=Homo sapiens OX=9606 GN=PHB1 PE=1 SV=1	8.879	MAPK, p53, stress
P11142	Heat shock cognate 71 kDa protein OS=Homo sapiens OX=9606 GN=HSPA8 PE=1 SV=1	7.815	stress
**P09429**	**High mobility group protein B1 OS=Homo sapiens OX=9606 GN=HMGB1 PE=1 SV=3**	**7.228**	**DNA damage repair, PI3K/AKT, JAK/STAT and NF-κB**
P04792	Heat shock protein beta-1 OS=Homo sapiens OX=9606 GN=HSPB1 PE=1 SV=2	7.133	stress
P05089	Arginase-1 OS=Homo sapiens OX=9606 GN=ARG1 PE=1 SV=2	6.563	metabolism, immune
P245	Elongation factor 1-beta OS=Homo sapiens OX=9606	6.53	translation, metabolism, stress

34	GN=EEF1B2 PE=1 SV=3	8	
Q9U	Mitochondrial dicarboxylate carrier OS=Homo sapiens OX=9606		
BX3	GN=SLC25A10 PE=1 SV=2	6.52	metabolism, ferroptosis, apoptosis
P840	60S ribosomal protein L19 OS=Homo sapiens OX=9606	6.46	
98	GN=RPL19 PE=1 SV=1	2	stress
P699	Hemoglobin subunit alpha OS=Homo sapiens OX=9606	6.33	
05	GN=HBA2 PE=1 SV=2	9	stress, metabolism
P110	Endoplasmic reticulum chaperone BiP OS=Homo sapiens	5.01	
21	OX=9606 GN=HSPA5 PE=1 SV=2	4	stress
P181	60S ribosomal protein L7 OS=Homo sapiens OX=9606	4.87	
24	GN=RPL7 PE=1 SV=1	8	stress, ferroptosis
Q085	Desmocollin-1 OS=Homo sapiens OX=9606 GN=DSC1 PE=1	4.87	
54	SV=2	4	β-catenin, autophagy, metabolism
P257	Proteasome subunit alpha type-3 OS=Homo sapiens OX=9606	4.84	
88	GN=PSMA3 PE=1 SV=2	9	stress, metabolism, AMPK
P007	Coagulation factor X OS=Homo sapiens OX=9606 GN=F10	4.59	
42	PE=1 SV=2	4	immune, PAR-1-dependent signaling
Q9Y5	Signal recognition particle receptor subunit beta OS=Homo	4.54	
M8	sapiens OX=9606 GN=SRPRB PE=1 SV=3	5	NF-κB, stress
Q151		4.03	
49	Plectin OS=Homo sapiens OX=9606 GN=PLEC PE=1 SV=3	9	MAPK/ERK, apoptosis
Q8W	MIT domain-containing protein 1 OS=Homo sapiens OX=9606		
V92	GN=MITD1 PE=1 SV=1	3.98	cell fate, AKT, ERK
P051	ADP/ATP translocase 2 OS=Homo sapiens OX=9606	3.68	
41	GN=SLC25A5 PE=1 SV=7	6	PI3K/AKT, ERK
P155	NAD(P)H dehydrogenase [quinone] 1 OS=Homo sapiens	3.61	
59	OX=9606 GN=NQO1 PE=1 SV=1	9	stress, metabolism, p53, PI3K/AKT
Q9N	Calmodulin-like protein 5 OS=Homo sapiens OX=9606	3.55	
ZT1	GN=CALML5 PE=1 SV=2	6	epidermal differentiation
P213		3.48	develpoment, DNA damage repair,
33	Filamin-A OS=Homo sapiens OX=9606 GN=FLNA PE=1 SV=4	3	Wnt/β-catenin
P122	ADP/ATP translocase 3 OS=Homo sapiens OX=9606	3.47	
36	GN=SLC25A6 PE=1 SV=4	8	development, metabolism
Q9H0	Magnesium transporter protein 1 OS=Homo sapiens OX=9606	3.39	MAPK, cell cycle, Wnt/β-catenin,
U3	GN=MAGT1 PE=1 SV=1	6	development
P301	Protein disulfide-isomerase A3 OS=Homo sapiens OX=9606		metabolism, DNA damage repair,
01	GN=PDIA3 PE=1 SV=4	3.37	stress
P007	Coagulation factor IX OS=Homo sapiens OX=9606 GN=F9	3.35	
40	PE=1 SV=2	1	chemoresistance, senescence, immune
Q9Y5	Cytosolic Fe-S cluster assembly factor NUBP2 OS=Homo	3.30	
Y2	sapiens OX=9606 GN=NUBP2 PE=1 SV=1	4	cell fate
P386	Stress-70 protein, mitochondrial OS=Homo sapiens OX=9606	3.21	
46	GN=HSPA9 PE=1 SV=2	2	stress, cell proliferation, p53
P064	Prothymosin alpha OS=Homo sapiens OX=9606 GN=PTMA	3.14	
54	PE=1 SV=2	2	apopotosis, stress, necrosis
Q6U			Wnt/β-catenin, MEK/ERK,
WP8	Suprabasin OS=Homo sapiens OX=9606 GN=SBSN PE=1 SV=2	3.12	chemoresistance
P381	Electron transfer flavoprotein subunit beta OS=Homo sapiens	3.04	
17	OX=9606 GN=ETFB PE=1 SV=3	8	metabolism, TGF-β
